# Tape Worm

**Published:** 1890-01

**Authors:** W. A. Rape


### TAPE WORM.

DR. W. A. RAPE, of Ballinger, sends the JOURNAL some interesting notes of his experience with this troublesome parasite. We present below his favorite prescription, one which the Doctor says has proved most useful in the treatment. He has tried many remedies and by preference gives now:

℞. Chloroform ʒss; to be given in some bland, acceptable vehicle, to be followed in half an hour by

℞. Ol. Tiglii Gtt. iv.

Ol. Ricini ℥ss.

Glycerine ℥ss.

Ag. destil ad ℥ii.

M. Sig. Give two teaspoonsful three hours apart regularly till the worm is expelled. After which give salicylic acid in small doses every three or four hours during the day for three or four days; being careful to keep the bowels open. The patient should fast for twenty-four hours previous to beginning the treatment. The Doctor says the tape worm is caused by the introduction into the stomach, of meat improperly prepared; that it is "a fact, based upon scientific investigation, that the Tænia S. is nothing more nor less than the product of the characteristic germ found in fresh meats," which finds a suitable nidus for its development and growth in the alimentary tract.

We presume the object of the salicylic acid after the expulsion of the worm, is to destroy any undeveloped germs that may remain behind.

Dr. Rape says he has treated a number of cases successfully as above, and asks for the views and experience of our readers through the JOURNAL. We will be pleased to have a discussion of the subject, and the JOURNAL is open to receive the views of the profession.

